# Alterations of Cerebral Hemodynamics and Network Properties Induced by Newsvendor Problem in the Human Prefrontal Cortex

**DOI:** 10.3389/fnhum.2020.598502

**Published:** 2021-01-15

**Authors:** Hashini Wanniarachchi, Yan Lang, Xinlong Wang, Tyrell Pruitt, Sridhar Nerur, Kay-Yut Chen, Hanli Liu

**Affiliations:** ^1^Department of Bioengineering, University of Texas at Arlington, Arlington, TX, United States; ^2^Department of Information Systems and Operations Management, University of Texas at Arlington, Arlington, TX, United States

**Keywords:** functional near infrared spectroscopy, risk decision-making, graph theory, brain network, newsvendor problem

## Abstract

While many publications have reported brain hemodynamic responses to decision-making under various conditions of risk, no inventory management scenarios, such as the newsvendor problem (NP), have been investigated in conjunction with neuroimaging. In this study, we hypothesized (I) that NP stimulates the dorsolateral prefrontal cortex (DLPFC) and the orbitofrontal cortex (OFC) joined with frontal polar area (FPA) significantly in the human brain, and (II) that local brain network properties are increased when a person transits from rest to the NP decision-making phase. A 77-channel functional near infrared spectroscopy (fNIRS) system with wide field-of-view (FOV) was employed to measure frontal cerebral hemodynamics in response to NP in 27 healthy human subjects. NP-induced changes in oxy-hemoglobin concentration, Δ[HbO], were investigated using a general linear model (GLM) and graph theory analysis (GTA). Significant activation induced by NP was shown in both DLPFC and OFC+FPA across all subjects. Specifically, higher risk NP with low-profit margins (LM) activated left-DLPFC but deactivated right-DLPFC in 14 subjects, while lower risk NP with high-profit margins (HM) stimulated both DLPFC and OFC+FPA in 13 subjects. The local efficiency, clustering coefficient, and path length of the network metrics were significantly enhanced under NP decision making. In summary, multi-channel fNIRS enabled us to identify DLPFC and OFC+FPA as key cortical regions of brain activations when subjects were making inventory-management risk decisions. We demonstrated that challenging NP resulted in the deactivation within right-DLPFC due to higher levels of stress. Also, local brain network properties were increased when a person transitioned from the rest phase to the NP decision-making phase.

## Introduction

Neuroeconomics is an emerging field that integrates economic theories with neuroscience to enhance the understanding of how the human brain makes decisions under different business conditions (Hsu et al., 2005). The “behavioral perspective in decision-making” is common in business research. For example, studies in management science have found that psychological factors, particularly attitudes toward risks and rewards, are essential drivers of business decisions. In general, business research usually assesses cognitive and psychological processes indirectly via human-subject experiments, surveys, and/or real-world observations. On the other hand, functional neuroimaging is a unique tool that allows researchers to “peek into the black box” and gather data directly from pertinent regions of the brain while subjects are engaged in making a decision. Advanced neuroimaging technologies, such as multi-channel electroencephalography (EEG) and functional magnetic resonance imaging (fMRI), are potentially able to provide clues to the underlying cognitive processes that previously were assumed to be the determinants of human decision-making.

However, neuroeconomics with neuroimaging is still in its infancy because of many challenges (Glimcher et al., 2008; Xue et al., 2010). For example, the fMRI measurement settings are very different from naturalistic environments, limiting accurate explanatory power for real-life decision-making processes. Also, it is uncertain “whether neuroimaging can provide theories for economists or whether economic theories can provide frameworks for neuroscience” (Xue et al., 2010). Thus, the goal of this study was to link neuroeconomics with neuroimaging in general and with functional near-infrared spectroscopy (fNIRS) in particular to better understand the behavioral perspective in decision-making in business research. The objective of this study was to quantify alterations of cerebral hemodynamics and network properties induced by the newsvendor problem (NP) in the human prefrontal cortex using a 77-channel fNIRS system.

Multi-channel fNIRS is a portable and non-invasive imaging technique that measures cortical hemodynamic activities in the human brain. It quantifies the cerebral concentration changes of oxygenated hemoglobin (Δ[HbO]) and deoxygenated hemoglobin (Δ[Hb]) with the high temporal resolution based on the alteration of optical absorption and scattering of near-infrared (NIR) light propagating through the human brain. The temporal and spatial features of Δ[HbO] and Δ[Hb] are then used as biomarkers of neuronal activations (Boas et al., 2004; Cazzell et al., 2012). In the last two decades, fNIRS has gained popularity and has been recognized as a non-invasive tool to functionally image brain activations and diagnose brain diseases (Benzion et al., 2008; Boas et al., 2014). Compared to fMRI, fNIRS is cost-effective and less sensitive to motion artifacts, has less restriction on body movement or confinement, and has a higher temporal resolution. All these features make it easier to use in a task-oriented, more-naturalistic experimental environment.

While many publications have reported brain activities in response to risk decision-making under varying conditions of risk, few studies have focused on inventory management scenarios, such as the NP, in conjunction with fNIRS techniques. NP has been widely used in management science (Gaspars-Wieloch, 2017) and refers to a prevalent business decision-making scenario in which an individual must balance between potential loss and waste to achieve maximum expected profit. The typical scenario is that of a store manager deciding the number of units of products to stock when the number of customers is uncertain (Gaspars-Wieloch, 2017). Too little stock leads to potential loss of sales; too much stock leads to potential waste. Risky decisions have to be made when stocking the inventory for profit under arbitrary requirements. The specialty of NP is to holistically consider business management scenarios, not just one element, for the best decision-making outcome. The NP setting is a corner stone inventory management scenario that reflects challenges that managers may encounter in practice. Kremer et al. showed that business decision-making, particularly in the newsvendor setting, is sensitive to context (Mirko et al., 2010). That is, the same problem when framed using different contexts often results in significantly different decision-making behaviors (Platt and Huettel, 2008). Hence, the right context is a crucial part of any research design that endeavors to explicate business decision-making behaviors. This study aims to fill the void in the extant literature by using fNIRS to map human brain activations or deactivations and cortical network changes caused by NP.

Graph theory analysis (GTA) is an analysis method that has been developed to examine large-scale complex brain networks (Bullmore and Sporns, 2009; Rubinov and Sporns, 2010; Braun et al., 2012; Kim et al., 2013; Liao et al., 2013). It can provide an easy and yet powerful mathematical means to characterize the topological properties of the brain networks (Sporns et al., 2005; Pavlopoulos et al., 2011; Vecchio et al., 2017). In particular, a few recent studies have combined GTA with channel-wise fNIRS, and have revealed the topological organization and architecture of large-scale, resting-state human brain cortical networks (Niu et al., 2012; Niu and He, 2014; Li et al., 2018). Furthermore, since the human brain is a dynamic organ, numerous alterations take place in the brain at fast time scales. Neuronal alterations occur in a millisecond scale while cerebral blood flow changes within seconds. Thus, dynamic brain connectivity and networks characterize the time-dependent neurophysiological processes that are associated with prompt decision making and cognition (Telesford et al., 2016; Hu et al., 2019). Recent literature has reported that dynamic connectivity can be quantified for time ranges in 15–120 s, and that a minimum temporal window for calculating connectivity is 30 s (Thompson and Fransson, 2015; Telesford et al., 2016; Farahani et al., 2019). While this 30-s duration is not a strict requirement, the longer the time window is, the less specific or dynamic the information gained.

This study also applied GTA to investigate dynamic brain networks when the human brain switched from resting state to dynamic task-based state. In particular, we performed our analysis using a dynamic connectivity approach: We took three of the decision trials and concatenated them to make or satisfy a reasonable time duration (~30 s) for dynamic connectivity calculation. Moreover, we took a three-trial moving window overlapping one trial for both decision-making and rest phase to achieve adequate time resolution for each subject.

Specifically, to examine dynamic activities of the human brain in response to the NP tasks, we formulated two hypotheses: Hypothesis I was that NP stimulates both dorsolateral prefrontal cortex (DLPFC) and orbitofrontal cortex (OFC) significantly in the human brain, and that a more challenging NP scenario results in the deactivation of right-DLPFC (R-DLPFC) in addition to activation of left-DLPFC (L-DLPFC). Hypothesis II was that the local efficiency (Eloc), cluster coefficient (Cp), and path length (Lp) of the human brain network are increased when a person switches from resting phase to the NP decision-making phase. To test these two hypotheses, we designed and incorporated the NP protocol using a computer-based platform with simultaneous 77-channel fNIRS data acquisition.

## Materials and Methods

### Participants

A total of 27 subjects (20 males and seven females; 23 ± 5 years of age) participated in the study. They were randomly assigned to two experimental groups with different risk levels. The subjects were included in the study if they met the following criteria: belonged to either sex, were from any ethnic background, and were between 18 and 40 years of age. The subjects were excluded only if they (1) were diagnosed with a psychiatric disorder, (2) had a history of a neurological condition or severe brain injury or violent behavior, (3) had a history of prior institutionalization or imprisonment, and (4) were currently under any medicine or drug. The study protocol complied with all applicable federal guidelines and was approved by the institutional review board (IRB) of the University of Texas at Arlington. Informed consent was obtained from every subject who participated in the experiment.

### NP Protocol Design

The experimental protocol design was based on the Newsvendor Problem (Gaspars-Wieloch, 2017), where a news vendor must decide how many newspapers to buy each day at the wholesale price and sell at the retail price. This NP problem has five major characteristics: (1) the demands are uncertain but from a known distribution; (2) the decision must be taken for every period; (3) there is a cost for ordering too many items; (4) the number of items ordered at each time is called order quantity, which must be decided for the inventory by the subject in each trial; and (5) each trial is independent. According to the NP model (Schweitzer and Cachon, 2000; Benzion et al., 2008; Gaspars-Wieloch, 2017), under the condition that the order quantity (*q)* is larger than the unknown demand (*D*), the final profit (π) can be calculated as a function of *q* and *D* by Equation (1) (Schweitzer and Cachon, 2000), as shown below:

(1) $$\begin{array}{r} \pi \left(q,D\right)=p\text{ min}\left(q,D\right)-c\;q \end{array}$$

where *c* is the cost, and *p* is the price to sell.

Based on the conditions listed above, the experimental protocol was designed with two independent treatments, namely, high-profit margin (HM) (*c* < < *p*) and low-profit margin (LM), (*c* < *p*). Table 1 shows the details of the design for the two different treatments used in our study. In the HM treatment, the price to sell, *p*, was designed to be $32 while the cost was only $8, all of which resulted in a lower risk of losing profits. On the other hand, in the LM treatment, while retaining p's value of $32, the cost was raised to $24, leading to a higher risk of losing profits. The demand was kept unknown until the participant made his or her decision by typing the order quantity between 0 and 300 per trial. Then, the given demand was randomly generated from a uniform distribution between 0 and 300 with a mean of 150. The history of demands from previous trials was visible to the participant after each trial, based on which the participant could decide for the current trial. There were 40 trials in total in each experiment. The selling price and cost were kept constant and known for each HM and LM treatment.

**Table 1:** NP protocol summary.

**Treatment**	**High-profit margin (HM)**	**Low-profit margin (LM)**
Price (p)	$32	$32
Cost (c)	$8	$24
Demand distribution	Uniform [0 300]	Uniform [0 300]

### NP Protocol Implementation

**Figure 1:**
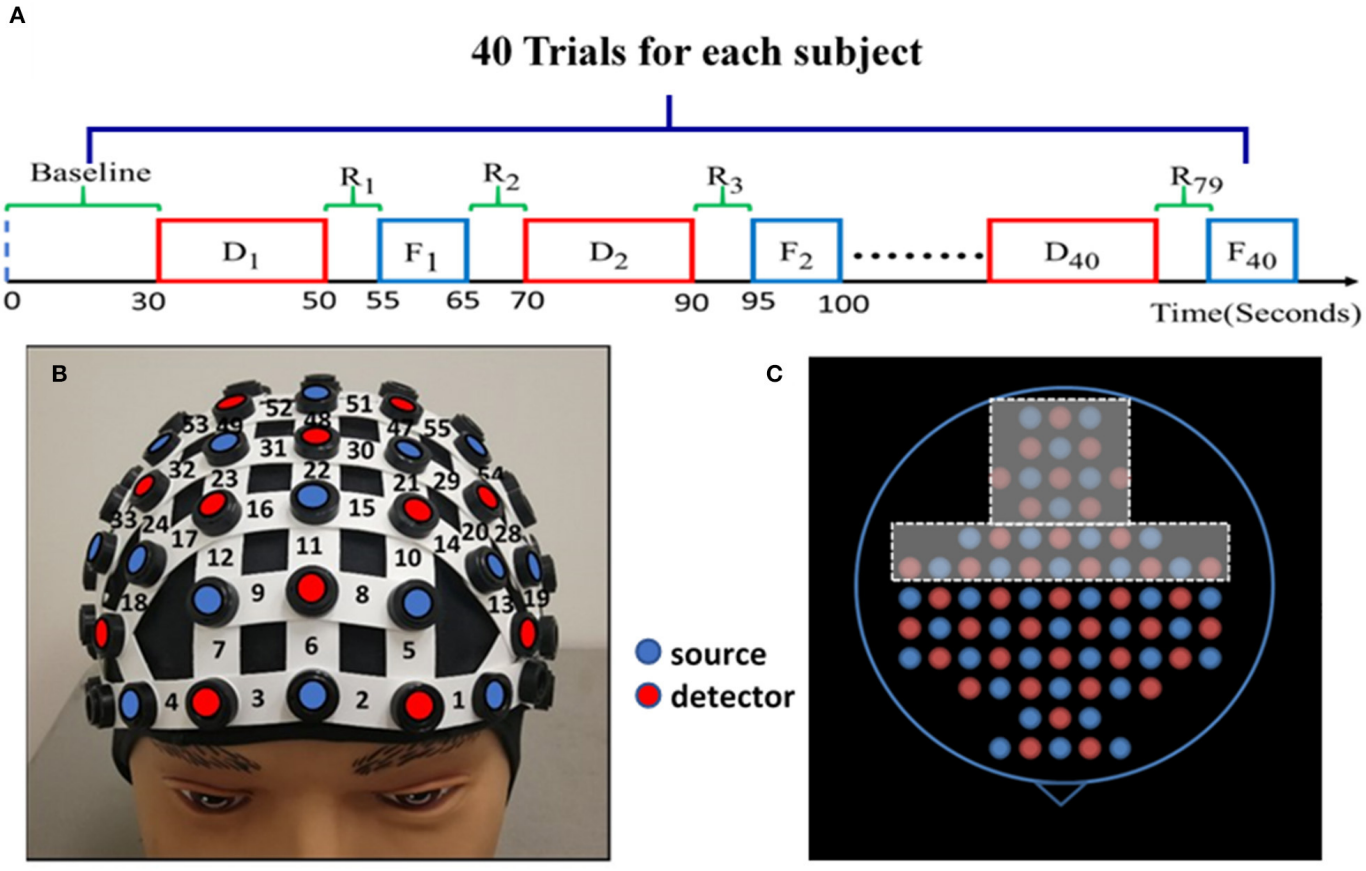
Experimental protocol and setup. **(A)** The NP experimental protocol consists of a 30-s initial baseline and 40 trials. Each trial has four phases: up to 20-s NP decision task (D_i_ ), 5-s rest (R_2i−1_), 10-s feedback (F_i_ ), and 5-s rest (R_2i_ ) again before starting another block/trial for D_i+1_ trial. (where i = 1, 2, 3, …, 40) Each block lasts about 30–40 s. **(B)** A demonstration of the fNIRS optode-setting helmet on a dummy head, holding 25 pairs of light sources (red dots) and 23 detectors (blue dots) with channel numbers marked covering the pre-frontal and frontal regions. **(C)** The entire optode layout for the whole-head helmet; the holes within the dashed frames were not used to hold any optode in this study while the other holes are color-matched to those shown in **(B)** on the forehead.

Subjects were randomly divided into either the HM treatment (*n* = 13) or LM treatment (*n* = 14) group before the experiment. One entire experiment consisted of a 30-s baseline and 40 blocks corresponding to 40 trials of NP tasks, as shown in Figure 1A. The 30-s “baseline” was needed to acquire the baseline of cerebral hemodynamic functions of each subject. Each block contained one NP trial, and each NP trial entailed four phases: decision, rest, feedback, and rest. The “decision” phase lasted for a maximum of 20 s, during which each subject was asked to decide the order quantity given such visible information as price, cost, and demand distribution range for either the HM or LM group. The subject was instructed to enter the quantity in a text box on the screen within the 20-s maximal period. If the subject did not enter anything within the 20 s, the program automatically entered the order quantity to be “0.” In general, decision-making durations were not the same for each subject over the trials.

After the decision phase, the screen shifted to a 5-s “rest” phase, followed by a 10-s “feedback” phase. Within this 10-s period, the subject was shown a summary table listing the price, cost, demand distribution, profit/loss, and the cumulative profits/losses that were made after every trial. Then the protocol proceeded to the next trial following another 5-s “rest” period. All trials of either LM or HM session consisted of the same setting during the decision phase. All the profit/loss details were stored along with the corresponding time stamps.

The NP trials were presented on a laptop computer in the form of a game. The subjects were directed to use both hands during the experiment, one hand to press the spacebar on the keyboard for making an event stamp for fNIRS, and the other to enter the order quantity for the corresponding trial. Before each experiment, each subject was provided information on how to play the NP game and given a 5-trial practice session to get familiar with the experimental protocol.

### fNIRS Experiment Setup and Optode Projection on a Human Cortex Template

A continuous-wave, multi-channel fNIRS system (LABNIRS, Shimadzu Corp., Kyoto, Japan) was employed in this study. As reported before (Cacola et al., 2018), a customized, wide field-of-view, 77-channel layout incorporating 25 sets of laser transmitters and 23 light receivers was used to cover the area from the prefrontal cortex to the sensorimotor cortex. Figure 1B demonstrates how a whole-head helmet facilitates all optical optodes to be attached to a human head. Figure 1C shows a top view of the source and detector layout with only its frontal portion being used in this study. The distance between the nearest source and detector optodes was 3 cm, resulting in a detection depth of 1.5–2 cm under the scalp. The helmet firmly and steadily held all the source and detector fibers on each subject's head; the data sampling frequency was 12.82 Hz. Spatial co-registration measurements were taken using a 3D digitizer (FASTRACK, Polhemus, Colchester, VT, USA). Montreal Neurological Institute (MNI) coordinates for each source and detector location were calculated using the statistical parametric mapping NIRS-SPM software (Ye et al., 2009), providing projected locations of channels on a human cortex template and corresponding Brodmann areas (BAs).

The frontal sinus makes it a challenge to image OFC—a unique region of the prefrontal cortex—accurately using fMRI. Similarly, fNIRS can reach OFC partially through recordings right on top of the eyebrows. To make sure that our fNIRS was able to acquire signals from OFC, we identified and listed in Table 2 the anatomical registrations of channels 1 to 4 (see Figure 1B) generated by NIRS-SPM (Ye et al., 2009) according to the optode locations (Hu et al., 2019), as an example. Specifically, this table shows two major BAs (BAs 10 and 11) and respective brain areas (i.e., frontopolar area (FPA) and OFC) that were covered by each of the four channels as well as the percentage of FPA and OFC covered by each channel. Since we could not separate recorded Δ[HbO] signals from OFC and FPA, they were grouped together and noted as OFC+FPA in the following sections.

**Table 2:** NIRS SPM output for anatomical registration of optodes.

**Channel #**	**BAs**	**Percentage of BA covered by the listed channel**
01	10–FPA	0.54
01	11–OFC	0.46
02	10–FPA	0.90
02	11–OFC	0.10
03	10–FPA	0.76
03	11–OFC	0.24
04	10–FPA	0.51
04	11–OFC	0.33
04	46-DLPFC	0.09
04	47-inferior prefrontal gyrus	0.07

### Data Analysis

#### Behavioral Analysis

The NP protocol generated two types of behavioral scores: (1) the decision-making (reaction) time and (2) the profit/loss score that each subject made for each trial. These two parameters were averaged across 40 trials for each subject for each of HM (*n* = 13) and LM (*n* = 14) groups. Then, the grand-averaged reaction time and profit score over each group were quantified and pooled with box plots to quantify their respective distributions. Statistical testing was next performed using a two-sample *t*-test for both averaged reaction time and profit score to identify a significant difference between the HM and LM groups.

#### fNIRS Data Preprocessing

The raw outputs of the fNIRS system were time-dependent optical intensities at three wavelengths (i.e., 780 nm, 805 nm, and 830 nm), alterations of which were affected by the changes of hemoglobin concentrations. A band-pass filter of 0.01–0.2 Hz was applied to remove artifacts from cardiac pulses (~0.8–1.2 Hz), respiration (~0.2–0.3 Hz) (Erdogan et al., 2014), muscle movements, and systemic drifts. Next, the modified Beer-Lambert Law was used to convert the recorded optical intensities at 780 nm and 830 nm into concentration changes of oxygenated hemoglobin (Δ[HbO]) and deoxygenated hemoglobin (Δ[Hb]) (Boas et al., 2004; Nguyen et al., 2018). Then, each time series of Δ[HbO] and Δ[Hb] was baseline calibrated by subtracting its temporal average to remove any potential drift during the baseline recording. Moreover, to further remove systemic, physiological variations contained in the scalp and skull, we subtracted the signal spatially averaged across all 77 channels to remove the global noise (Cacola et al., 2018). Also, the channels close to the superficial temporal artery were excluded from the analysis to prevent signal mixing with patterns from arteries (Smielewski et al., 1997; Oldag et al., 2012; Urquhart et al., 2020). Afterward, the pre-processed data were used for further analysis. The data analysis was performed in two steps: (1) NP-evoked Δ[HbO] activations based on a general linear model (GLM) and (2) NP-evoked alterations in brain connectivity based on GTA.

#### Activation Maps Based on the General Linear Model

GLM is a mathematical model popularly applied in fMRI (Calhoun et al., 2001, 2004) and fNIRS (Lin et al., 2014; Tian et al., 2014) to estimate amplitudes of hemodynamic changes at different brain regions in response to a variety of tasks. In our study, a preprocessed time series of Δ[HbO] per channel was used as the input in the GLM analysis using MATLAB. In the process, the designed temporal matrix was generated with a boxcar function, reflecting the NP task blocks convoluted with a hemodynamic response function (HRF) (Schroeter et al., 2004). For each NP experiment (with HM or LM treatment), two regressors were assumed for the decision and feedback phase, while the data analysis focused only on the decision phase. Equation (2) demonstrates the GLM model, where “*y*” is the time series; *x*_1_ and *x*_2_ represent the designed temporal matrix corresponding to the decision and feedback phase, respectively; β_1_ and β_2_ are the amplitudes of Δ[HbO] for the two phases; and “*e*” is the fitting error term:

(2) $$\begin{array}{r} y=\beta_{1}x_{1}+\beta_{2}x_{2}+e. \end{array}$$

With this model, the activation amplitudes (β_1_ and β_2_) were fitted with a regression algorithm between measured Δ[HbO] time series and the temporal design matrix using a weighted least square method (Tian et al., 2014). Such regression processes were carried out for each channel, resulting in an array of 77 fitted values for each of β_1_ and β_2_ relative to the baselines for each human subject. In this study, we focused on β_1_ only since it reflected Δ[HbO] responses to the decision phase.

Next, β_1_ values were averaged across the subjects for each of the 77 channels, which permitted us to form topographic maps on the frontal cortex at the group-level. Topographic ΔHbO maps were generated using easytopo 2.0 (Tian et al., 2014). Then we utilized NIRS-SPM (Gaspars-Wieloch, 2017) to identify and illustrate several key cortical areas, namely, DLPFC (BAs 9 and 46) and OFC+FPA (BAs 10 and 11), on the topographic maps, with or without considering HM or LM treatment.

Due to a relatively small sample size, we decided to take a region-wise (not channel-wise) statistical analysis, focusing on three prefrontal regions of interest (pf-ROIs), R-DLPFC, L-DLPFC, and OFC+FPA. Specifically, β_1_ values of Δ[HbO] within each of the three pf-ROIs were summed for each subject and then averaged at group levels with or without considering any decision-difficulty effect. One-sample and two-sample *t*-tests were performed to test our hypothesis I. In particular, a one-sample *t*-test (against the mean of zero baselines) was taken on area-summed β_1_ values for each of the pf-ROIs at the group-level from all 27 subjects without considering the degree of decision difficulty. This step allowed us to identify any of the three pf-ROIs that were significantly evoked by NP, as the main effect, while the subjects made risky NP decisions. Then, a two-sample *t*-test was performed on area-summed β_1_ values for each of the pf-ROIs between the HM (*n* = 13) and LM (*n* = 14) groups to examine whether a more challenging NP (i.e., with LM tasks) created a significant difference of brain response in any of three pf-ROIs with respect to easy HM tasks. All activation-related statistical tests were performed at a significance level of α = 0.05 (with the Bonferroni correction).

#### Correlation Between Brain Activation and Behavioral Performance

To better understand the association of brain activation with performance outcome, we analyzed correlations between performance scores achieved in NP tasks and area-summed β_1_ values of Δ[HbO] within each of the three pf-ROIs. The performance was measured using the average profit each subject made. For each of pf-ROIs, each pair of individual profit and respective summed β_1_ value from each subject was plotted and used to determine their linear correlation. The linear correlation analysis was performed at the significance level of α = 0.05 without separating LM and HM treatment.

#### Applying GTA to Assess Network Properties in the Brain

Graph theory is a renowned mathematical model to study characteristics of a network system (Erdös, 1959; Bondy and Murty, 1976), and it has been applied to investigate resting state functional connectivity of the human brain as measured with fMRI, Electroencephalography (EEG), and Magnetoencephalographic (MEG) (Salvador et al., 2005; Achard et al., 2006; Bassett et al., 2006; Iturria-Medina et al., 2008), as well as fNIRS (Niu et al., 2012; Niu and He, 2014; Li et al., 2018). A recent study demonstrated that based on GTA, brain networks were altered when the human brain transitioned from resting state to task-evoked states (Hu et al., 2019). In this study, we also applied GTA to primarily focus on how brain networks were altered from resting state to the decision-making state in a dynamic context.

**Figure 2:**
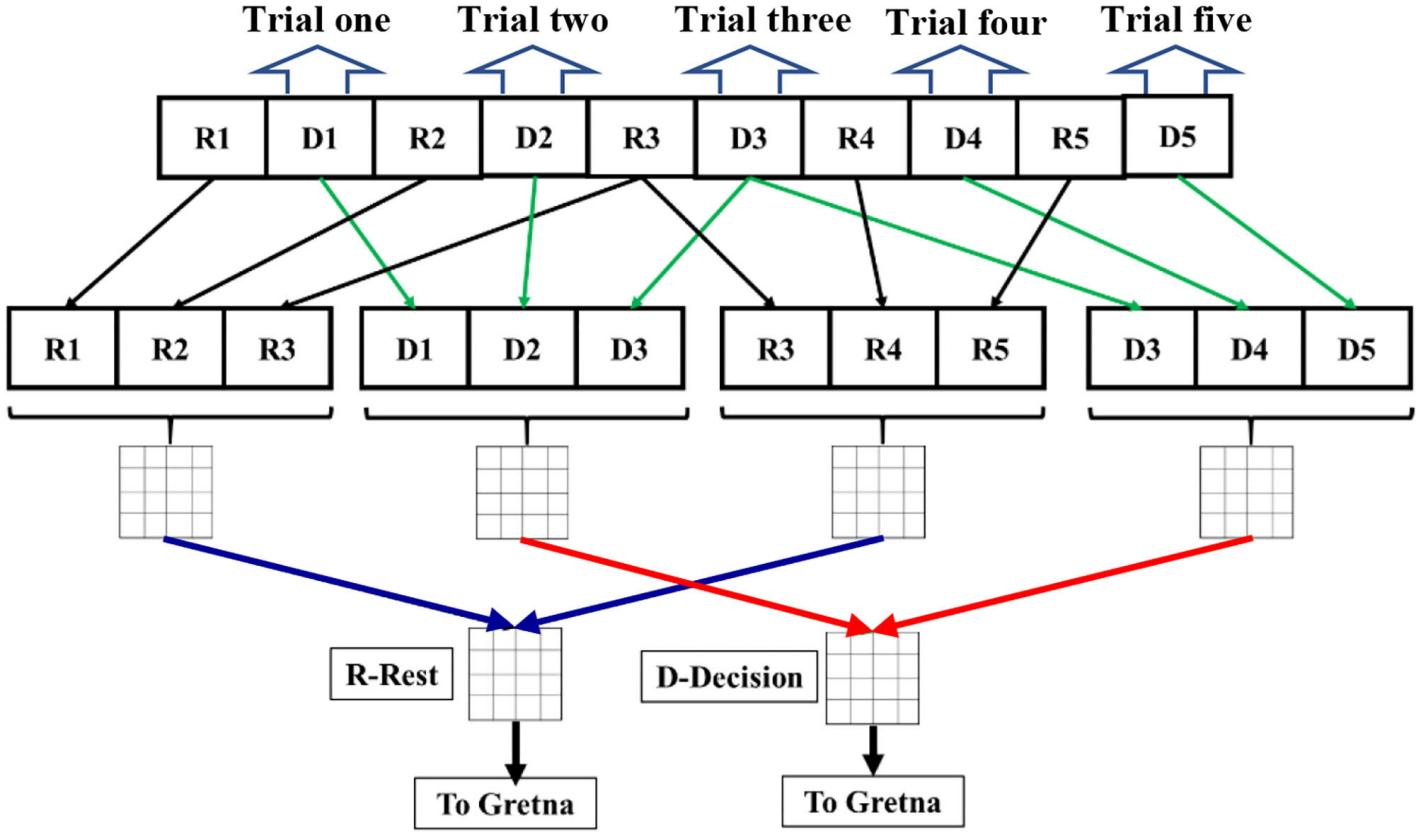
A schematic flow chart for dynamic graph theory analysis from the 1st trial to 5th trial. Ri denotes the rest period during the ith trial. Di denotes the decision period during the ith period. Rest (black lines) and decision periods (green lines) of three trials were extracted and concatenated into two time series separately in a three-trial moving window overlapping one trial (see that R3 and D3 are used twice for three-trial windows). Each concatenated time series were used to calculate PCC for the dynamic connectivity at each respective time. Then, correlation matrices from multiple three-trial windows were averaged for both rest (blue lines) and decision (red lines) periods, respectively. The final averaged correlation matrices (labeled as R-Rest and D-Decision) underwent Fischer r-to-z transform to calculate network parameters in GRETNA or Gretna for each subject.

Our analysis based on GTA was performed following similar steps to those in (Niu et al., 2012; Niu and He, 2014; Cai et al., 2018; Li et al., 2018), as follows: (1) The processed Δ[HbO] time series for all channels were analyzed to create Pearson's correlation coefficient (PCC) matrices in the rest and decision-making phases. Only positive correlations were considered for calculations. The reason for avoiding negative correlations is that there is lack of biological explanation for negative correlations (Niu et al., 2012; Niu and He, 2014). Nodes were defined as channels and edges were dependent on normalized Pearson's correlation strength. (2) Rest and decision periods of three trials (see Figure 1A) were extracted and concatenated into two time series separately. (3) Since the average time for a decision trial was about 10 s, we took three of the decision trials and concatenated them to make a reasonable time duration for dynamic connectivity quantification. The three-trial moving window was applied, overlapping one trial between the two consecutive windows to create multiple concatenated time series (see Figure 2). This operation resulted in multiple PCC matrices for both rest and decision phases, respectively (see figure legend of Figure 2). Only the first 20 trials were included for the connectivity calculations to avoid impacts from fatigue and stress. (4) The PCC matrices for all concatenated time series were converted to z-values by Fisher's r-to-z transformation to improve normality, resulting in a 77 × 77 z-value functional connectivity matrix Zij for each subject, where i, j = 1, 2, … 77. (5) All three-trial-derived, Fisher-transformed Z matrices were averaged to become two respective matrices for both rest and task phases for each subject. (6) These two matrices were entered as inputs in GRaph thEoreTical Network Analysis (GRETNA) (Wang et al., 2015) to construct the functional brain network for each subject. (7) Within GRETNA, we chose the sparsity, S, as the threshold criterion, where S is the number of current existing edges divided by the total possible number of edges in the current matrix in a network.

We selected and quantified three global topological properties/metrics to study network patterns with a range of S level (S; 0.05 < S < 0.50; increment = 0.05) (Achard and Bullmore, 2007), as follows: (1) local efficiency, Eloc, that describes how efficient the communication is between the first neighbors of *i* when *i* is removed; (2) clustering coefficient, Cp, that measures network segregation; and (3) path length, Lp, that is the average of the shortest path length between all pairs of nodes. Detailed definitions and explanations of these network metrics can be found in Cai et al. (2018). These metrics were chosen primarily because they are network parameters popularly reported in literature (Iturria-Medina et al., 2008; Tamburro et al., 2020), Furthermore, these metrics focus on local network connections and may reflect more direct linkage or association with local brain activations.

All seven steps were repeatedly performed on all 27 subjects, followed by statistical comparisons with two-sample *t*-tests between the resting and dynamic decision-making phase for each of the three network-metrics within 0.05 < S < 0.50 at the significance level of α = 0.01.

## Results

### Behavioral Results

**Figure 3:**
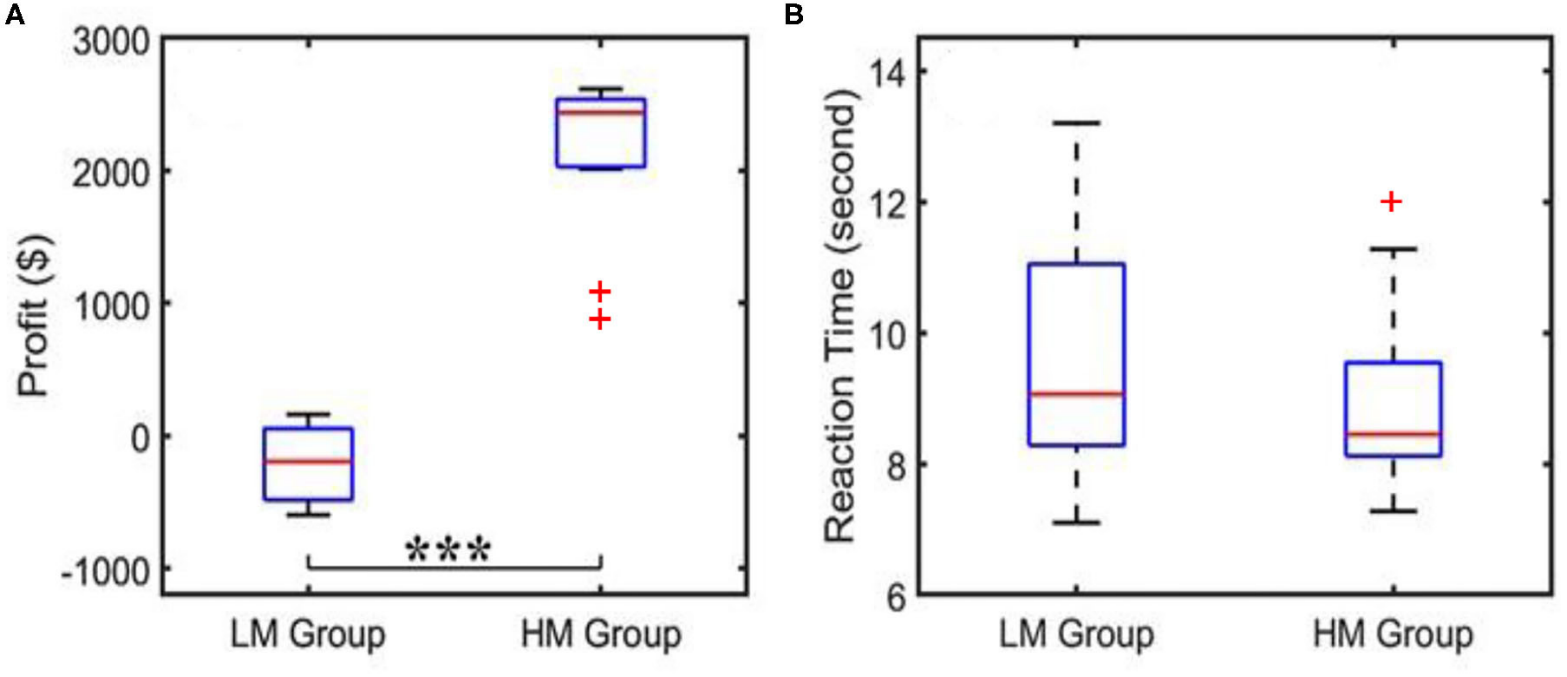
Behavioral scores showing **(A)** an average of profit per trial and **(B)** an average reaction or decision-making time acquired from the LM and HM groups. “***”indicates the statistical significance between the two groups at *p* < 0.001 (*p* = 1.9 × 10^−13^). The reaction time did not show a significant difference. Outliers are marked by “+”.

When the subjects played NP games, their profit/loss scores and the reaction times per trial were recorded, as well as the average scores and average decision-making times over 40 trials. The corresponding results for both LM and HM groups are shown in Figure 3. Figure 3A shows that the profit score averaged over the LM group was nearly zero, whereas the HM group gained a mean profit of $2,600, with a large significant difference in profit between the two groups as determined by a two-sample *t*-test. Figure 3B shows that the subjects under the LM protocol spent an average of 9 s to make the risk decision while the HM group needed an average of 7 s to complete the task, which was not significantly shorter by a two-sample *t*-test. Results shown in Figure 3A were expected because the HM group had a much easier scenario to make good profit, as compared to the LM treatment.

### Brain Activation Evoked by NP Determined With GLM

As a result of GLM analysis, the group-averaged channel-wise β_1_ values were generated based on hemodynamic changes, Δ[HbO], when subjects performed the NP-based tasks. Respective statistical tests aided us in identifying significant activations on the prefrontal cortex in response to the decision-making tasks. Two statistical analyses were performed to test whether: (1) any of three pf-ROIs was significantly evoked by the NP with respect to the baseline regardless of treatment (HM or LM) types, and (2) more challenging NP tasks (i.e., LM) created a significant difference in brain activation or deactivation in DLPFC or OFC +FPA regions with respect to less challenging NP conditions.

**Figure 4:**
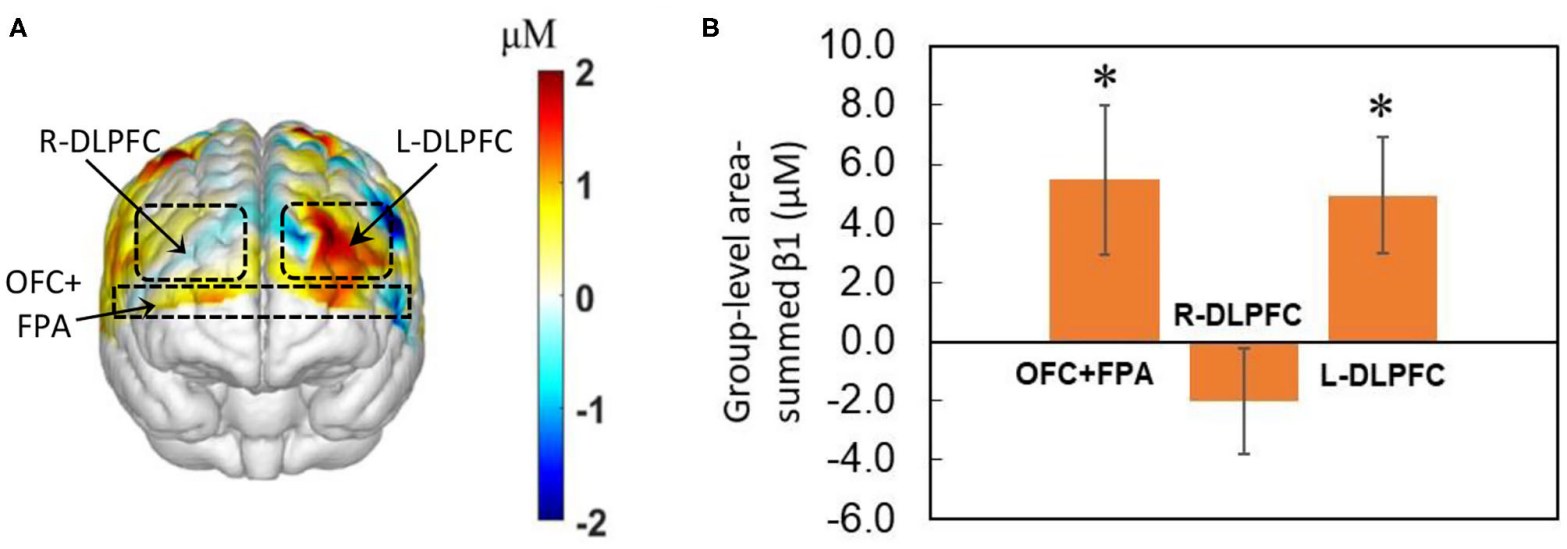
Topographic β_1_ maps in response to brain stimulations by the 40-trial NP from all subjects (*n* = 27) without considering the treatment level. **(A)** Averaged topographic beta (or β_1_) map derived from Δ[HbO] during the decision-making phase. Dashed lines mark estimated regions of the three pf-ROIs: R-DLPDC, L-DLPDC, and OFC+FPA. **(B)** Group-averaged, area-summed β_1_ values for decision-making relative to baseline for OFC+FPA (*p* < 0.015), R-DLPFC and L-DLPFC (*p* < 0.008), respectively. “*” denotes *p* < 0.05 (corrected). Error bars: standard error of the mean.

We obtained the front view of a topographic Δ[HbO]-derived beta (i.e., β_1_) map, as shown in Figure 4A, averaged over the 40 decision-making trials from all 27 human subjects regardless of HM or LM treatment. The topographic map illustrates activated cortical areas identified by the output of NIRS-SPM (Ye et al., 2009) corresponding to the co-registration readings. Figure 4B presents the corresponding group-level area-summed β_1_ values from respective three pf-ROIs. The statistical analysis based on the one-sample *t*-test illustrates that NP tasks significantly (*p* < 0.05, corrected) activated Δ[HbO] in both the OFC+PFA and L-DLPFC, but not in the R-DLPFC, with respect to the baseline.

**Figure 5:**
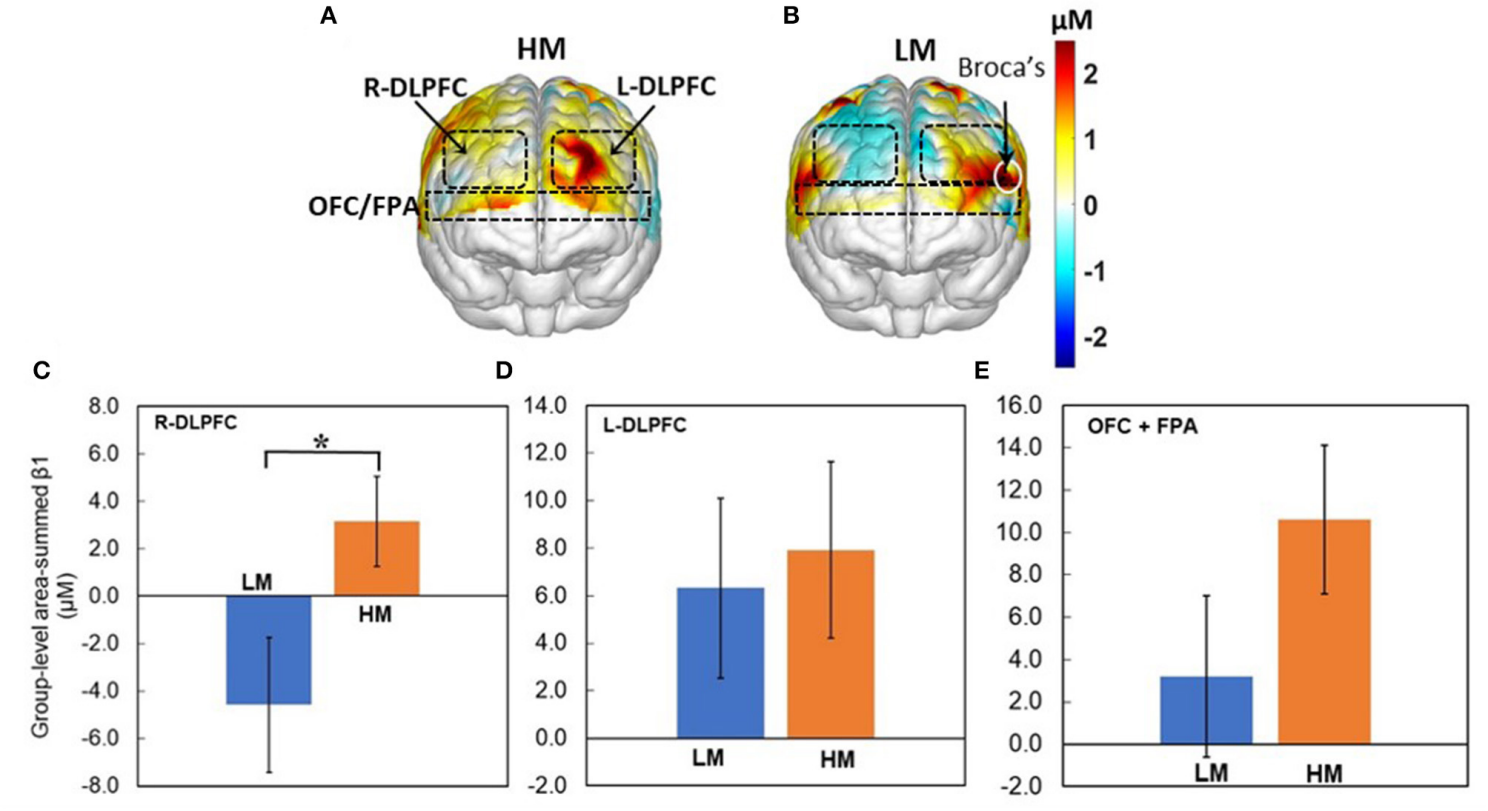
Topographic β_1_ maps of Δ[HbO] in response to the 40-trial NP from groups with **(A)** HM (*n* = 13) and **(B)** LM (*n* = 14) treatment. Group-averaged, area-summed β_1_ values from groups with HM and LM decision-making tasks in **(C)** the R-DLPFC (*p* < 0.015), **(D)** the L-DLPFC, and **(E)** the OFC+FPA, respectively. “*” denotes *p* < 0.05 (corrected). Error bars: standard error of the mean.

Next, Figures 5A,B show topographic Δ[HbO] β_1_ maps from 13 and 14 human subjects in response to HM or LM treatment, respectively. It is clear that both HM and LM activated L-DLPFC, while LM appeared to deactivate R-DLPFC. Figures 5C–E plot respective group-level area-summed β_1_ values from three pf-ROIs, R-DLPDC, L-DLPDC, and OFC+FPA, respectively, with statistically significant difference marked between Δ[HbO] values under HM and LM treatment. These figures reveal that within the R-DLPFC, more challenging NP with LM significantly (*p* < 0.05; corrected) deactivated Δ[HbO] than the easier NP tasks with HM, and that both HM and LM tasks evoked brain activations but without significant differences between them in each of L-DLPFC and OFC+FPA. All these observations supported Hypothesis I that NP stimulates both DLPFC and OFC significantly in the human brain, and that more challenging NP results in significant deactivation of Δ[HbO] in the R-DLPFC.

### Correlation Between Prefrontal Cortex Activation and Behavioral Performance

**Figure 6:**
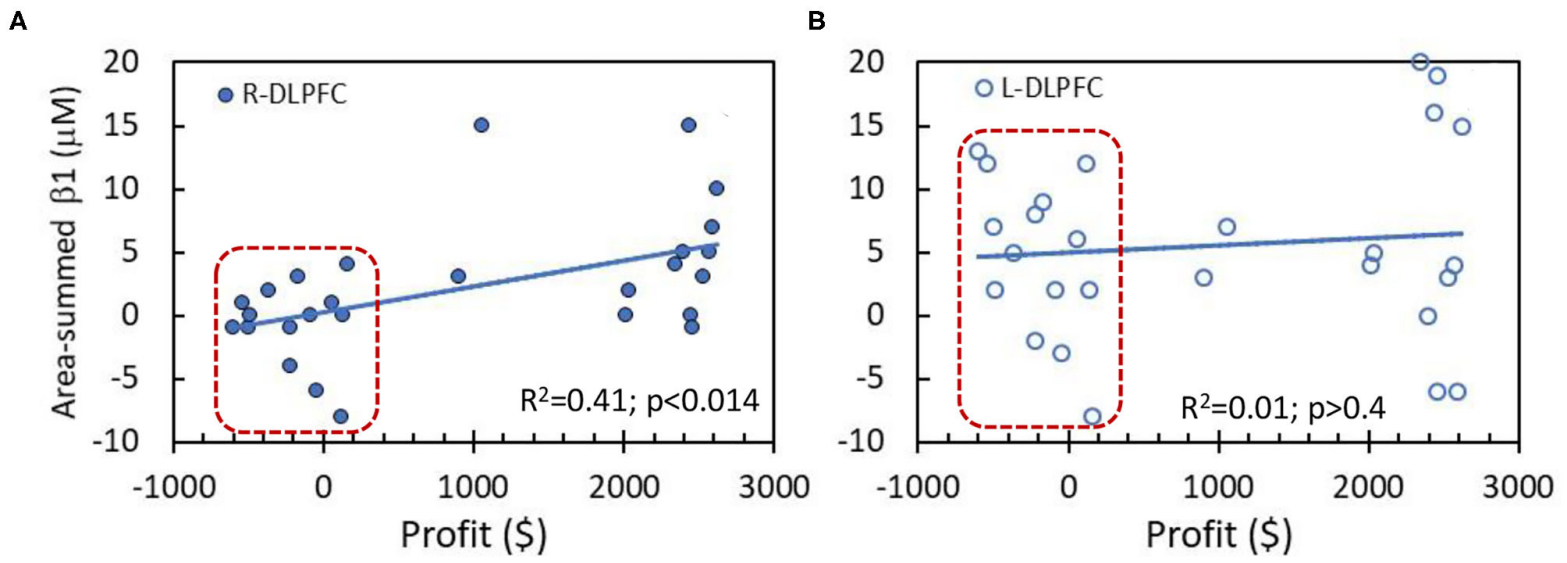
Correlations between behavior performance (i.e., profit values) and area-summed β_1_ values of Δ[HbO] from the entire region of **(A)** R-DLPFC and **(B)** L-DLPFC. The data within the red boxes were derived from the LM group (*n* = 14). The solid lines are a linear fit across all the subjects (*n* = 27 without separating HM and LM tasks) with *R*^2^ value of 0.41 and 0.01, respectively, for both DLPFC areas. A significant linear correlation exists only for R-DLPFC at *p* < 0.014.

The correlation analysis was performed between the performance scores (i.e., profit values) in NP tasks and area-summed β_1_ values of Δ[HbO] in two key prefrontal cortical areas, namely, R-DLPFC and L-DLPFC, together for both HM and LM tasks, as shown in Figures 6A,B. A linear correlation (Figure 6A) between the profit and summed β_1_ values is statistically significant (*p* < 0.014) in the R-DLPFC in spite of the scattered appearance of the data. On the other hand, no similar correlation pattern is seen in the R-DLPFC (Figure 6B). After close inspection of the data, we noticed two separate clusters, one of which is outlined by a red box in each of the two panels. Indeed, the data within the boxes were derived from the LM group (*n* = 14) with much lower profit values as compared to those from the HM group (*n* = 13).

### Brain Network Changes Induced by NP Analyzed by GTA

**Figure 7:**
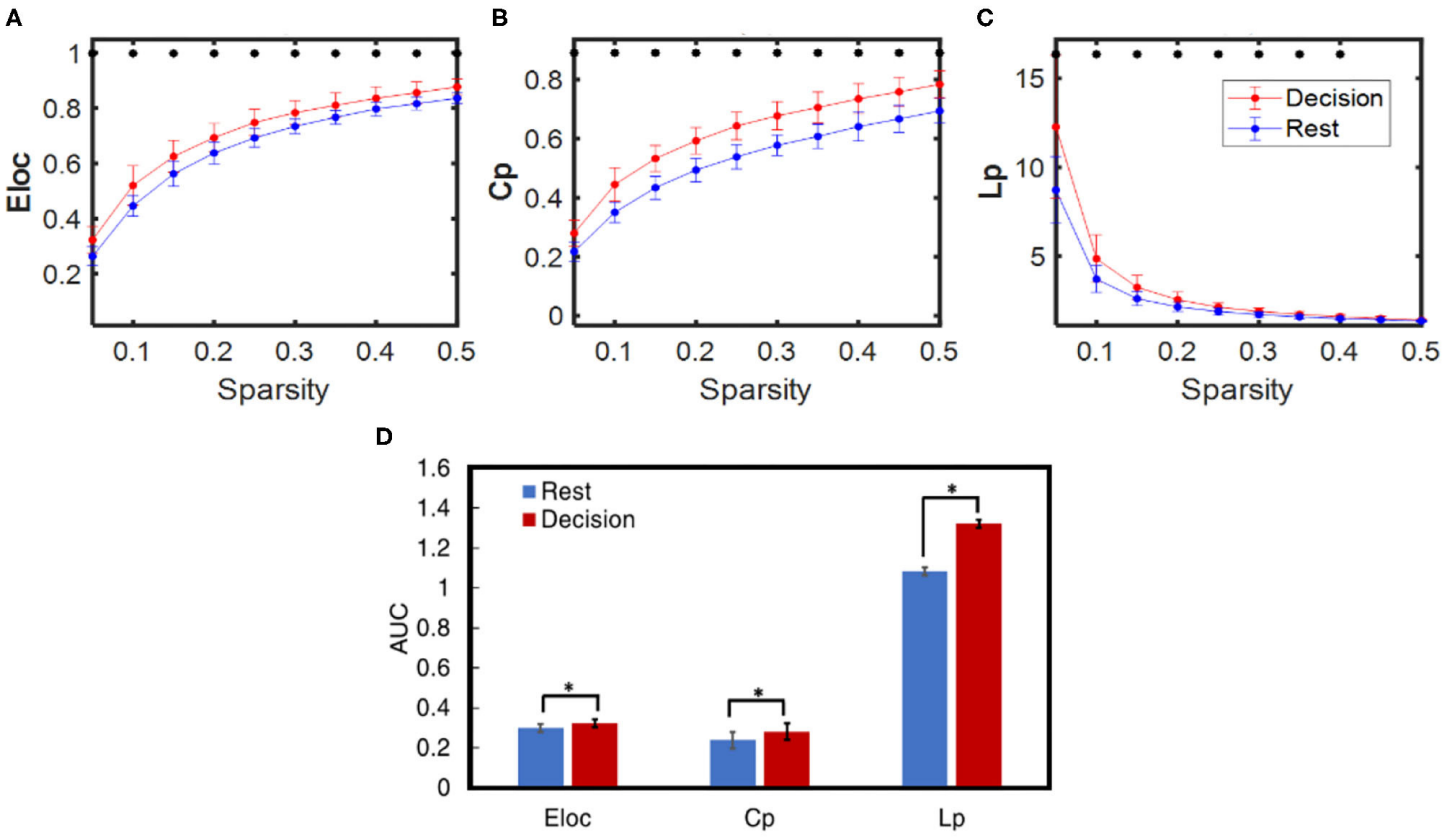
Comparison of averaged dynamic functional network properties derived from GTA during NP decision (red) and rest (blue) phases for all subjects (*n* = 27): **(A)** Eloc, **(B)** Cp, and **(C)** Lp considering sparsity ranging from 0.05 to 0.5, and **(D)** three respective network metrics quantified by the area under the curve (AUC) within the sparsity of 0.05–0.5 for the decision (red) and rest (blue) phases. In all panels, the error bars are the standard deviation. The * indicates the significant difference between the decision and rest phase for each network metric at *p* < 0.01.

Dynamic brain network properties during the resting and decision phases were obtained based on GTA, yielding three selected global network properties: Eloc, Cp, and Lp for sparsity between 0.05 and 0.5. Consequently, Figures 7A–C illustrate respective network properties averaged over all subjects (*n* = 27) including both HM and LM tasks. Two-sample *t*-tests were performed for each of the three network-metrics within 0.05 < S < 0.50 between the rest vs. decision phase regardless of LM or HM at *p* < 0.05 and (ii) between LM and HM at *p* < 0.05. Regarding the statistical test between the rest and decision phase, Figure 7 marks that the NP decision tasks significantly increased Eloc, Cp, and Lp compared to the rest phase for almost all of the sparsity range (0.05–0.4 thresholds). The same statistical conclusions hold consistently when we took an alternative approach by comparing the integrated differences (i.e., area under the curve, AUC) across sparsities between 0.05 and 0.5, as shown in Figure 7D. However, the other two-sample *t*-test between HM and LM groups did not show any significant difference at any sparsity threshold for any of the global network properties.

## Discussion

In a complex and turbulent business environment, managers must make decisions that require complex trade-offs and risk considerations. Decision-making under risk is a complex cognitive process, requiring contribution and integration of actions from multiple regions of the human brain (Gold and Shadlen, 2007; Farb, 2013; Khani and Rainer, 2016). The scope of this study was to observe which cortical regions of the human frontal cortex are responsible for making risky decisions in a business context, as represented by the widely studied newsvendor problem. Hypothesis I was that NP stimulates both DLPFC and OFC+FPA significantly in the human cortex and that more challenging NP with LM treatment triggers more deactivation in DLPFC than HM treatment. Hypothesis II was that brain network increases its Eloc, Cp, and Lp when the human brain switched from rest state to the NP decision phase. To prove these hypotheses, we conducted 77-channel, wide field-of-view fNIRS measurements from 27 human subjects concurrently when they performed NP decision-making tasks. After performing data analyses and reviewing the results, we have learned several valuable lessons and gained scientific insights, as discussed below.

### Behavioral Outcomes Affected by NP Decision-Making Tasks

The behavioral scores (Figure 3) revealed how NP tasks with HM and LM affected subjects' performance in average profits and average decision-making time. During the LM treatment, it was difficult to earn positive rewards while the LM group took about the same time as the HM group to make the decision. In contrast, the HM group received much better and significant profits. These observations may imply that the subjects facing the LM challenge were under much more stress than those facing the HM condition, as the former group had a more challenging and risky, time-bound decision-making task that compelled them to think much harder and more carefully to gain profits.

### Brain Activation by NP Decision-Making Tasks

Our results (Figure 4) confirmed that both DLPFC and OFC+FPA regions, particularly L-DLPFC, played significant and critical roles in cognitive processing when subjects had to solve the NP regardless of difficulty levels. Our findings are consistent with several previous studies in the literature that found DLPFC responsible for risky decision-making (in non-business context) and cognitive control in terms of planning and working memory (Cazzell et al., 2012; Makwana and Hare, 2012; Huang et al., 2017; Farrar et al., 2018). Another significantly activated area observed in this study was the combination of partial FPA with partial OFC (i.e., BAs 10 and 11), which are also crucial parts of the brain involved in executive functions. Even though they are not a part of the reward pathway, there is evidence of their contribution to decision-making.

Moreover, a slight deactivation was observed in the left anterior temporal lobe. One of the studies comparing brain activation on fMRI-based semantic and non-semantic tasks has shown deactivation in the left anterior temporal lobe in response to non-semantic tasks while semantic tasks triggered activation. Also, left-lateralized activation is closely related to scripted words or generating speech. Our study was more related to a non-semantic aspect. Therefore, the deactivation observed in the left-temporal lobe can be attributed to the comprehension of written words when reading NP questions (Visser and Lambon Ralph, 2011; Humphreys et al., 2015).

### Brain Activation by Low-Risk (HM) NP Tasks

It is seen (Figure 5A) that the HM group presented prominent activation within the L-DLPFC when making low-risk NP decisions. This protocol scenario would be similar to a goal-oriented decision-making task in a less risky environment. Our observation on L-DLPFC is consistent with a few published reports. In an fMRI-based study where the subjects were given the task of “Tower of London,” a popular protocol based on planning, it was observed that the left DLPFC was significantly activated during the hierarchical goal state (Kaller et al., 2011). Another study reported significant DLPFC stimulations when the subjects decided to execute a right-hand movement to enter the order quantity on the computer keyboard (Hoshi and Tanji, 2004). Furthermore, we observed that OFC+FPA were activated during the HM NP tasks. Since OFC is suggested to be one of the significant components in the reward pathway (Bolla et al., 2003), the activation of partial OFC explains the anticipation for gains during the trials.

### Brain Activation by High-Risk (LM) NP Tasks

Compared to HM stimulation, several distinct features of the brain responses to LM stimulation (Figure 5B) are noted: (1) strong activation within the L-DLPFC and partial Broca's area, (2) apparent deactivation in the R-DLPFC, and (3) weak activation/deactivation in OFC + FPA. This set of tasks was more challenging than those with HM, so the subjects needed to pay more attention to the tasks and to control their emotions during the tasks. Since Broca's area has a function of modulating emotional response besides the language process, it is reasonable to observe certain activation in this cortical area in response to the LM tasks. Also, the observed R-DLPFC deactivation may be attributed to intense stress induced by more difficult decision-making challenges (Qin et al., 2009; Bogdanov and Schwabe, 2016). OFC is expected to have a strong response in this case because OFC plays a key role in decision-making involving reward, but we did not observe such activations in OFC. A plausible reason for this finding is that, as the more challenging NP tasks shifted the subjects' attention from a less-stressful reward phase to a more-stressful defying phase, the brain activations occurred in DLPFC rather than in OFC.

### Differences in Brain Activation Between High-Risk (LM) and Low-Risk (HM) NP Tasks

Two-sample *t*-tests on brain responses to LM and HM treatments revealed that LM tasks triggered significant deactivation within the R-DLPFC (Figure 5C). This observation is consistent with the results reported in several recent publications. For example, an fMRI study involving 27 subjects suggested that deactivation in R-DLPFC may occur due to acute stress, which weakens high-level cognitive functions such as working memory (Dias and Segraves, 1999). Also, a transcranial direct current stimulation (tDCS) based study with 120 participants showed that tDCS delivered on the R-DLPFC could prevent stress-induced working memory deficits (Qin et al., 2009; Bogdanov and Schwabe, 2016). In our study, the NP with the LM protocol was somewhat risky and a bit lengthy, subjecting the participants to higher levels of stress. Consequently, we observed clear and significant deactivation in r-DLPEC only during LM tasks.

### Correlation Between Brain Activation vs. Performance in NP Decision-Making

Figure 6A demonstrates a significant NP-evoked correlation between the NP decision-making outcome vs. the brain activation/deactivation in the R-DLPFC. The literature reports that R-DLPFC deactivation takes place when a subject experiences stress or fatigue (Qin et al., 2009; Bogdanov and Schwabe, 2016). Our results present consistent findings at the individual and group levels, respectively, as shown in Figures 5C, 6A. These two figures demonstrate that Δ[HbO] in the R-DLPFC is reduced when subjects faced difficult decisions while the corresponding Δ[HbO] increased significantly (*p* < 0.015) when they performed well in making easy decisions to make profits. On the other hand, L-DLPFC has shown significant increases in Δ[HbO] under both easy and hard decision-making conditions to win profits (Figure 5D), but without any significant correlation between the performance outcome vs. Δ[HbO] activation (Figure 6B). Overall, our results hinted that brain activation in the L-DLPFC is closely associated with risk decision-making regardless of the level of challenges, while brain activation or deactivation in the R-DLPFC is driven by the stress level when facing difficult decision tasks.

### Brain Network Alteration Caused by NP Decision-Making Tasks

Our GTA-derived analysis (Figure 7) demonstrated that, regardless of difficulty levels, NP-evoked decision-making tasks altered the global dynamic network properties of the human brain from those at rest. The observation of increases in Eloc, Cp, and Lp (in sparsity range of 0.05–0.5) caused by NP indicates that NP stimulated sub-region connections in the brain more locally, not globally.

This observation also implies that more communications among regional segregations and/or clusters take place to achieve decision-making tasks. Also, increases in Eloc and Cp (in sparsity range of 0.05–0.5) as well as Lp (in sparsity range of 0.05–0.4) by NP reveal that NP decision-making needs the brain to boost or enhance network segregation/clustering, which also leads to an increase of Lp and Eloc consistently (Hu et al., 2019). As evidenced, Figures 4A,B illustrate that only a few cortical regions (i.e., DLPFC and OFC+FPA), not the entire prefrontal cortex, were involved in the decision-making process, regardless of either LM or HM treatment. These results indirectly support the underlying reasoning of enhanced Eloc and Cp during NP tasks.

Graph theory provides the conceptual foundation to investigate the network properties behind brain fluctuations. Many of the graph theory-based analyses focus on resting state networks. However, there are a few investigations that explore dynamic network properties during a task phase. Tamburro et al. explored connectivity and efficiency of the brain networks during an endurance cycling task (Tamburro et al., 2020). The study discussed several aspects of global network parameters that could affect behavior. The increase in Eloc probably indicates the transition from resting state to a specific task state, which may require a wide range of information interchange related to new settings among all brain regions before task execution (Petsche et al., 1997; Pfurtscheller and Andrew, 1999). In this study, we observed Eloc and Cp increase during our decision phase with respect to the rest phase, indicating the transition from the resting state. Moreover, it was suggested that the reduction of global efficiency (Eg) is a probable sign of hard effort (Kong et al., 2015; Tamburro et al., 2020). In theory, Eg and Lp are highly anticorrelated, a reduction of Eg is equivalent to an increase of Lp. In our study, the primary objective for each subject was to make right decisions for winning profits, so each person had to make high-demanding decisions for profitable outcomes. Thus, it is expected that NP decision-making tasks would result in an increase in Lp.

### Link Between Functional Activation and Network Connectivity in the Human Brain

The human brain acts as one cohesive complex network, connecting all brain regions and sub-networks collectively into one intricate system. Brain network provides valuable information about the local and global functional connectivity (Petsche et al., 1997) and incorporates information and interaction capability (Bullmore and Sporns, 2009; Kong et al., 2015; Geng et al., 2017; Hu et al., 2019; Wanniarachchi et al., 2020). Graph theory has been used commonly to examine such brain networks at resting state. In the field of NIRS, task-driven brain activations and GTA-derived network connectivity are often studied separately (Niu et al., 2012; Niu and He, 2014; Li et al., 2018). A recent study revealed that resting-state properties and task-evoked networks in the human brain can have significant correlations with the activation of brain regions during tasks of executive functions (Hu et al., 2019). In our study, we demonstrated that the NP decision making not only triggered significantly the prefrontal activation of Δ[HbO] in DLPFC and OFC+FPA (Figure 4) but also increased significantly local efficiency, clustering coefficients, and path length (Figure 7). Our findings supported an association between NP-evoked brain activation and brain dynamic network metrics. Since cortical regions of DLPFC and OFC+FPA are involved for making NP decisions, we expect that localized increases in blood oxygenation (i.e., Δ[HbO]) are necessary for information processing and decision making. In the meantime, we also expect that NP decisions would perturb/change brain networks in these cortical regions dynamically with enhanced local network efficiency and clustering coefficients for delivering an optimal decision most effectively. Consequently, an increase of network clustering coefficient would lead to an increase in path length due to more information processing locally within a local network region. Putting both aspects of cortical activation and local network enhancement together, our findings exhibited that NP decision making triggered local cortical stimulations in DLPFC and OFC+FPA, which concurrently gave rise to increases in (i) efficient communication between network nodes (Eloc), (ii) network segregation (Cp), and (iii) average number of the shortest path length between all pairs of nodes within the given network.

### Summary of New Findings in This Study

While we have revealed and discussed multiple new findings and new knowledge learned in this study, it would be helpful to summarize several key results and novel aspects with respect to previous reports.

First, the specialty of NP is to consider whole business management scenarios, not just one element, for the best decision-making outcome. The NP setting is sensitive to context (Mirko et al., 2010), which often results in significantly different decision-making behaviors (Platt and Huettel, 2008). One novelty of this study was to utilize NP as the experimental protocol. Second, because of the novel NP protocol, we were able to create risk decision-making treatments under two distinct levels of stress, which permitted us to better associate regional cortical activities (i.e., L-DLPFC, R-DLPFC, and OFC+FPA) with profitable decision-making outcomes. Third, with this unique experimental design, we observed a linear correlation between Δ[HbO] activation vs. performance outcome in the R-DLPFC, but the same Δ[HbO] response would be highly deactivated under high stress. On the other hand, the L-DLPFC would be always activated during the NP decision-making regardless of any stress. Fourth, GTA can be used to analyze dynamic functional connectivity when the human brain switches from the rest to a decision-making phase by implementing a three-trial, 60-s moving window to achieve adequate time resolution for each subject. Fifth, with the multi-trial moving window approach, we were able to analyze and determine significant increases of brain network metrics, such as Eloc, Cp, and Lp. All these parameters reflect the enhancement of local activation, communication, and information exchange within local cortical areas during the dynamic transition in the human brain from a resting phase to a decision phase. Last, further exploration or validation is needed to quantify direct association between task-based network properties vs. performance outcome in a larger sample size.

### Limitations of the Study and Future Work

First, due to limited coverage and spatial resolution, we could not separate Δ[HbO] signals from OFC and FPA; more optodes to be placed on the forehead can be a solution for this drawback. Second, even though the focused and detected areas of brain activation by NP were DLPFC and OFC+FPA at the cortical level, multiple other brain regions beyond the cortex were involved in the complex NP decision-making processes, but they are challenging to be reached by fNIRS. Third, since the 27 subjects had to be split into the LM and HM groups, each sample size was statistically small. It is appropriate in future studies to validate the findings of this study with a larger sample size. Last, the dynamic brain network properties or metrics were obtained using PCC with a relatively short period of time for both rest (~15 s) and NP (~ maximum of 60 s) task phases. Such a short period is perhaps not adequate to provide stable or physiologically meaningful results (Geng et al., 2017). More rigorous and appropriate algorithms for quantifying dynamic functional connectivity of the human brain at both rest and task-evoked phases need to be further explored and applied in the future.

## Conclusion

In conclusion, this study showed that NP-based decision-making stimulated vital brain areas, such as DLPFC and OFC, for high-level cognitive functions based on 77-channel, wide field-of-view, hemodynamic measurements with fNIRS from 27 human control subjects. The study observed that there were multiple regions activated and deactivated in response to the tasks. Explicitly, DLPFC and OFC+FPA were significantly evoked by NP tasks vs. baseline regardless of treatment types. Significant deactivation in R-DLPFC was observed and attributed to the challenging stress created by the LM with respect to HM. Furthermore, NP decision-making altered global brain network properties from the resting phase such that Eloc, Cp, and Lp were all increased. All these alterations in network properties together enhanced better communications among sub-regions or local segmentations to achieve NP-evoked decision tasks. Overall, this study supported our hypotheses: (1) that NP stimulates both DLPFC and OFC+FPA significantly in the human prefrontal cortex, and that more challenging NP results in deactivation in the right-DLPFC in addition to activation of left-DLPFC, and (2) that the local efficiency, cluster coeffcient, and path length of the brain network are increased when a person switches from resting phase to the NP decision-making phase.

## Data Availability Statement

The raw data supporting the conclusions of this article will be made available by the authors to anyone requesting them, without undue reservation.

## Ethics Statement

The studies involving human participants were reviewed and approved by the Institutional Review Board of the University of Texas at Arlington, Arlington, TX, 76019. The patients/participants provided their written informed consent to participate in this study.

## Author Contributions

HW performed the experiment, analyzed the data, interpreted the results, and prepared the manuscript. YL recruited human participants, implemented the newsvendor experiment protocols, and coordinated the experiment. TP assisted with experiment setup design, participated data collection, and discussed results. XW analyzed the data, discussed the results, and participated in manuscript revision. SN and K-YC designed the newsvendor experiment, discussed the results, and revised the manuscript. HL initiated and supervised the study, discussed and interpreted the results, as well as reviewed, and revised the manuscript. All authors contributed to the article and approved the submitted version.

## Conflict of Interest

K-YC has a potential research conflict of interest due to a financial interest with companies Hewlett-Packard Enterprise, Boostr, and DecisionNext. A management plan has been created to preserve objectivity in research in accordance with UTA policy. The remaining authors declare that the research was conducted in the absence of any commercial or financial relationships that could be construed as a potential conflict of interest.

## References

[B1] Achard S. Bullmore E. (2007). Efficiency and cost of economical brain functional networks. PLoS Comput. Biol. 3:e17 10.1371/journal.pcbi.0030017 17274684 PMC1794324

[B2] Achard S. Salvador R. Whitcher B. Suckling J. Bullmore E. (2006). A resilient, low-frequency, small-world human brain functional network with highly connected association cortical hubs. J. Neurosci. 26, 63–72. 10.1523/JNEUROSCI.3874-05.2006 16399673 PMC6674299

[B3] Bassett D. S. Meyer-Lindenberg A. Achard S. Duke T. Bullmore E. (2006). Adaptive reconfiguration of fractal small-world human brain functional networks. Proc. Natl. Acad. Sci. U.S.A. 103, 19518–19423. 10.1073/pnas.0606005103 17159150 PMC1838565

[B4] Benzion U. Cohen Y. Peled R. Shavit T. (2008). Decision-making and the newsvendor problem: an experimental study. J. Oper. Res. Soc. 59, 1281–1287. 10.1057/palgrave.jors.2602470

[B5] Boas D. A. Dale A. M. Franceschini M. A. (2004). Diffuse optical imaging of brain activation: approaches to optimizing image sensitivity, resolution, and accuracy. Neuroimage 23(Suppl. 1), S275–S288. 10.1016/j.neuroimage.2004.07.011 15501097

[B6] Boas D. A. Elwell C. E. Ferrari M. Taga G. (2014). Twenty years of functional near-infrared spectroscopy: introduction for the special issue. Neuroimage 85(Pt. 1), 1–5. 10.1016/j.neuroimage.2013.11.033 24321364

[B7] Bogdanov M. Schwabe L. (2016). Transcranial stimulation of the dorsolateral prefrontal cortex prevents stress-induced working memory deficits. J. Neurosci. 36, 1429–1437. 10.1523/JNEUROSCI.3687-15.2016 26818528 PMC6604824

[B8] Bolla K. I. Eldreth D. A. London E. D. Kiehl K. A. Mouratidis M. Contoreggi C.. (2003). Orbitofrontal cortex dysfunction in abstinent cocaine abusers performing a decision-making task. Neuroimage 19, 1085–1094. 10.1016/S1053-8119(03)00113-7 12880834 PMC2767245

[B9] Bondy J. A. Murty U. S. R. (1976). Graph Theory with Applications. London, UK: Macmillan.

[B10] Braun U. Plichta M. M. Esslinger C. Sauer C. Haddad L. Grimm O.. (2012). Test-retest reliability of resting-state connectivity network characteristics using fMRI and graph theoretical measures. Neuroimage 59, 1404–1412. 10.1016/j.neuroimage.2011.08.044 21888983

[B11] Bullmore E. Sporns O. (2009). Complex brain networks: graph theoretical analysis of structural and functional systems. Nat. Rev. Neurosci. 10, 186–198. 10.1038/nrn2575 19190637

[B12] Cacola P. Getchell N. Srinivasan D. Alexandrakis G. Liu H. (2018). Cortical activity in fine-motor tasks in children with developmental coordination disorder: a preliminary fNIRS study. Int. J. Dev. Neurosci. 65, 83–90. 10.1016/j.ijdevneu.2017.11.001 29126862

[B13] Cai L. Dong Q. Niu H. (2018). The development of functional network organization in early childhood and early adolescence: a resting-state fNIRS study. Dev. Cogn. Neurosci. 30, 223–235. 10.1016/j.dcn.2018.03.003 29631206 PMC6969083

[B14] Calhoun V. D. Adali T. McGinty V. B. Pekar J. J. Watson T. D. Pearlson G. D. (2001). fMRI activation in a visual-perception task: network of areas detected using the general linear model and independent components analysis. Neuroimage 14, 1080–1088. 10.1006/nimg.2001.0921 11697939

[B15] Calhoun V. D. Stevens M. C. Pearlson G. D. Kiehl K. A. (2004). fMRI analysis with the general linear model: removal of latency-induced amplitude bias by incorporation of hemodynamic derivative terms. Neuroimage 22, 252–257. 10.1016/j.neuroimage.2003.12.029 15110015

[B16] Cazzell M. Li L. Lin Z. J. Patel S. J. Liu H. (2012). Comparison of neural correlates of risk decision making between genders: an exploratory fNIRS study of the Balloon Analogue Risk Task (BART). Neuroimage 62, 1896–1911. 10.1016/j.neuroimage.2012.05.030 22634214

[B17] Dias E. C. Segraves M. A. (1999). Muscimol-induced inactivation of monkey frontal eye field: effects on visually and memory-guided saccades. J. Neurophysiol. 81, 2191–2214. 10.1152/jn.1999.81.5.2191 10322059

[B18] Erdogan S. B. Yücel M. A. Akin A. (2014). Analysis of task-evoked systemic interference in fNIRS measurements: Insights from fMRI. Neuroimage 87, 490–504. 10.1016/j.neuroimage.2013.10.024 24148922

[B19] Erdös P. (1959). Graph theory and probability. Canad. J. Math. 11, 34–38. 10.4153/CJM-1959-003-9

[B20] Farahani F. V. Karwowski W. Lighthall N. R. (2019). Application of graph theory for identifying connectivity patterns in human brain networks: a systematic review. Front. Neurosci. 13:585. 10.3389/fnins.2019.00585 31249501 PMC6582769

[B21] Farb N. A. S. (2013). Can neuroimaging inform economic theories of decision making. Neurosci. Neuroecon. 2013:1–10. 10.2147/NAN.S39339

[B22] Farrar D. C. Mian A. Z. Budson A. E. Moss M. B. Killiany R. J. (2018). Functional brain networks involved in decision-making under certain and uncertain conditions. Neuroradiology 60, 61–69. 10.1007/s00234-017-1949-1 29164280 PMC5798459

[B23] Gaspars-Wieloch H. (2017). Newsvendor problem under complete uncertainty: a case of innovative products. Cent. Eur. J. Oper. Res. 25, 561–585,. 10.1007/s10100-016-0458-3 28855846 PMC5556468

[B24] Geng S. Liu X. Biswal B. B. Niu H. (2017). Effect of resting-state fNIRS scanning duration on functional brain connectivity and graph theory metrics of brain network. Front. Neurosci. 11:392. 10.3389/fnins.2017.00392 28775676 PMC5517460

[B25] Glimcher P. W. Camerer C. Poldrack R. A. Fehr E. (2008). Neuroeconomics: Decision Making and the Brain. (Oxford: Academic Press).

[B26] Gold J. I. Shadlen M. N. (2007). The neural basis of decision making. Annu. Rev. Neurosci. 30, 535–574. 10.1146/annurev.neuro.29.051605.113038 17600525

[B27] Hoshi E. Tanji J. (2004). Area-selective neuronal activity in the dorsolateral prefrontal cortex for information retrieval and action planning. J. Neurophysiol. 91, 2707–2722. 10.1152/jn.00904.2003 14749313

[B28] Hsu M. Bhatt M. Adolphs R. Tranel D. Camerer C. F. (2005). Neural systems responding to degrees of uncertainty in human decision-making. Science 310, 1680–1683. 10.1126/science.1115327 16339445

[B29] Hu Z. Zhang J. Zhang L. Xiang Y. T. Yuan Z. (2019). Linking brain activation to topological organization in the frontal lobe as a synergistic indicator to characterize the difference between various cognitive processes of executive functions. Neurophotonics 6:025008. 10.1117/1.NPh.6.2.025008 31172018 PMC6537479

[B30] Huang D. Chen S. Wang S. Shi J. Ye H. Luo J.. (2017). Activation of the DLPFC reveals an asymmetric effect in risky decision making: evidence from a tDCS study. Front. Psychol. 8:38. 10.3389/fpsyg.2017.00038 28174549 PMC5258744

[B31] Humphreys G. F. Hoffman P. Visser M. Binney R. J. Lambon Ralph A. M. (2015). Establishing task- and modality-dependent dissociations between the semantic and default mode networks. Proc. Natl. Acad. Sci. U.S.A. 112, 7857–7862. 10.1073/pnas.1422760112 26056304 PMC4485123

[B32] Iturria-Medina Y. Sotero R. C. Canales-Rodríguez E. J. Alemán-Gómez Y. Melie-García L. (2008). Studying the human brain anatomical network via diffusion-weighted MRI and graph theory. NeuroImage 40, 1064–1076. 10.1016/j.neuroimage.2007.10.060 18272400

[B33] Kaller C. P. Rahm B. Spreer J. Weiller C. Unterrainer J. M. (2011). Dissociable contributions of left and right dorsolateral prefrontal cortex in planning. *Cereb*. Cortex 21, 307–317. 10.1093/cercor/bhq096 20522540

[B34] Khani A. Rainer G. (2016). Neural and neurochemical basis of reinforcement-guided decision making. J. Neurophysiol. 116, 724–741. 10.1152/jn.01113.2015 27226454

[B35] Kim D. J. Bolbecker A. R. Howell J. Rass O. Sporns O. Hetrick W. P.. (2013). Disturbed resting state EEG synchronization in bipolar disorder: a graph-theoretic analysis. NeuroImage Clin. 2, 414–423. 10.1016/j.nicl.2013.03.007 24179795 PMC3777715

[B36] Kong W. Lin W. Babiloni F. Hu S. Borghini G. (2015). Investigating driver fatigue versus alertness using the granger causality network. Sensors 15, 19181–19198. 10.3390/s150819181 26251909 PMC4570365

[B37] Li L. Babawale O. Yennu A. Trowbridge C. Hulla R. Gatchel R. J.. (2018). Whole-cortical graphical networks at wakeful rest in young and older adults revealed by functional near-infrared spectroscopy. Neurophotonics 5:035004. 10.1117/1.NPh.5.3.035004 30137882 PMC6063133

[B38] Liao X. H. Xia M. R. Xu T. Dai Z. J. Cao X. Y. Niu H. J.. (2013). Functional brain hubs and their test-retest reliability: a multiband resting-state functional MRI study. Neuroimage 83, 969–982. 10.1016/j.neuroimage.2013.07.058 23899725

[B39] Lin Z. J. Li L. Cazzell M. Liu H. (2014). Atlas-guided volumetric diffuse optical tomography enhanced by generalized linear model analysis to image risk decision-making responses in young adults. Hum. Brain Mapp. 35, 4249–4266. 10.1002/hbm.22459 24619964 PMC4282392

[B40] Makwana A. Hare T. (2012). Stop and be fair: DLPFC development contributes to social decision making. Neuron 73, 859–861. 10.1016/j.neuron.2012.02.010 22405198

[B41] Mirko K. Minner S. Van Wassenhove N. L. (2010). Do random errors explain newsvendor behavior. Manuf. Serv. Operat. Manag. 12, 673–681. 10.1287/msom.1100.0294

[B42] Nguyen T. Babawale O. Kim T. Jo H. J. Liu H. Kim J. G. (2018). Exploring brain functional connectivity in rest and sleep states: a fNIRS study. Sci. Rep. 8:16144. 10.1038/s41598-018-33439-2 30385843 PMC6212555

[B43] Niu H. He Y. (2014). Resting-state functional brain connectivity: lessons from functional near-infrared spectroscopy. Neuroscientist 20, 173–188. 10.1177/1073858413502707 24022325

[B44] Niu H. Wang J. Zhao T. Shu N. He Y. (2012). Revealing topological organization of human brain functional networks with resting-state functional near infrared spectroscopy. PLoS ONE 7:e45771. 10.1371/journal.pone.0045771 23029235 PMC3454388

[B45] Oldag A. Goertler M. Bertz A. K. Schreiber S. Stoppel C. Heinze H. J.. (2012). Assessment of cortical hemodynamics by multichannel near-infrared spectroscopy in steno-occlusive disease of the middle cerebral artery. Stroke 43, 2980–2985. 10.1161/STROKEAHA.112.656710 23091122

[B46] Pavlopoulos G. A. Secrier M. Moschopoulos C. N. Soldatos T. G. Kossida S. Aerts J.. (2011). Using graph theory to analyze biological networks. BioData Min. 4:10. 10.1186/1756-0381-4-10 21527005 PMC3101653

[B47] Petsche H. Kaplan S. von Stein A. Filz O. (1997). The possible meaning of the upper and lower alpha frequency ranges for cognitive and creative tasks. Int. J. Psychophysiol. 26, 77–97. 10.1016/S0167-8760(97)00757-5 9202996

[B48] Pfurtscheller G. Andrew C. (1999). Event-related changes of band power and coherence: methodology and interpretation. J. Clin. Neurophysiol. 16, 512–519. 10.1097/00004691-199911000-00003 10600019

[B49] Platt M. L. Huettel S. A. (2008). Risky business: the neuroeconomics of decision making under uncertainty. Nat. Neurosci. 11, 398–403. 10.1038/nn2062 18368046 PMC3065064

[B50] Qin S. Hermans E. J. van Marle H. J. F. Luo J. Fernández G. (2009). Acute psychological stress reduces working memory-related activity in the dorsolateral prefrontal cortex. Biol. Psychiatry 66, 25–32. 10.1016/j.biopsych.2009.03.006 19403118

[B51] Rubinov M. Sporns O. (2010). Complex network measures of brain connectivity: uses and interpretations. Neuroimage 52, 1059–1069. 10.1016/j.neuroimage.2009.10.003 19819337

[B52] Salvador R. Suckling J. Coleman M. R. Pickard J. D. Menon D. Bullmore E. (2005). Neurophysiological architecture of functional magnetic resonance images of human brain. Cereb. Cortex 15, 1332–1342. 10.1093/cercor/bhi016 15635061

[B53] Schroeter M. L. Bücheler M. M. Müller K. Uludag K Obrig H. Lohmann G.. (2004). Towards a standard analysis for functional near-infrared imaging. Neuroimage 21, 283–290. 10.1016/j.neuroimage.2003.09.054 14741666

[B54] Schweitzer M. E. Cachon G. P. (2000). Decision bias in the newsvendor problem with a known demand distribution: experimental evidence. Manage. Sci. 46, 404–420. 10.1287/mnsc.46.3.404.12070

[B55] Smielewski P. Czosnyka M. Pickard J. D. Kirkpatrick P. (1997). Clinical evaluation of near-infrared spectroscopy for testing cerebrovascular reactivity in patients with carotid artery disease. Stroke 28, 331–338. 10.1161/01.STR.28.2.331 9040685

[B56] Sporns O. Tononi G. Kötter R. (2005). The human connectome: a structural description of the human brain. PLoS Comput. Biol. 1:e42. 10.1371/journal.pcbi.0010042 16201007 PMC1239902

[B57] Tamburro G. di Fronso S. Robazza C. Bertollo M. Comani S. (2020). Modulation of brain functional connectivity and efficiency during an endurance cycling task: a source-level EEG and graph theory approach. Front. Hum. Neurosci. 14:243. 10.3389/fnhum.2020.00243 32733219 PMC7363938

[B58] Telesford Q. K. Lynall M. E. Vettel J. Miller M. B. Grafton S. T. Bassett D. S. (2016). Detection of functional brain network reconfiguration during task-driven cognitive states. Neuroimage 142, 198–210. 10.1016/j.neuroimage.2016.05.078 27261162 PMC5133201

[B59] Thompson W. H. Fransson P. (2015). The frequency dimension of fMRI dynamic connectivity: Network connectivity, functional hubs and integration in the resting brain. Neuroimage 121, 227–242. 10.1016/j.neuroimage.2015.07.022 26169321

[B60] Tian F. Yennu A. Smith-Osborne A. Gonzalez-Lima F. North C. S. Liu H. (2014). Prefrontal responses to digit span memory phases in patients with post-traumatic stress disorder (PTSD): a functional near infrared spectroscopy study. Neuroimage Clin. 4, 808–819. 10.1016/j.nicl.2014.05.005 24936431 PMC4055895

[B61] Urquhart E. L. Wang X. Liu H. Fadel P. J. Alexandrakis G. (2020). Differences in net information flow and dynamic connectivity metrics between physically active and inactive subjects measured by functional near-infrared spectroscopy (fNIRS) during a fatiguing handgrip task. Front. Neurosci. 14:167. 10.3389/fnins.2020.00167 32210748 PMC7076120

[B62] Vecchio F. Miraglia F. Maria Rossini P. (2017). Connectome: graph theory application in functional brain network architecture. Clin. Neurophysiol. Pract. 2, 206–213. 10.1016/j.cnp.2017.09.003 30214997 PMC6123924

[B63] Visser M. Lambon Ralph A. M. (2011). Differential contributions of bilateral ventral anterior temporal lobe and left anterior superior temporal gyrus to semantic processes. J. Cogn. Neurosci. 23, 3121–3131. 10.1162/jocn_a_00007 21391767

[B64] Wang J. Wang X. Xia M. Liao X. Evans A. He Y. (2015). GRETNA: a graph theoretical network analysis toolbox for imaging connectomics. Front. Hum. Neurosci. 9:458 10.3389/fnhum.2015.00458 26175682 PMC4485071

[B65] Wanniarachchi H. Lang Y. Wang X. Nerur S. Chen K.-Y. Liu H. (2020). Neural correlates of newsvendor-based decision making in the human brain: an exploratory study to link neuroeconomics with neuroimaging using fNIRS. bioRxiv [Preprint]. 10.1101/2020.02.08.940197

[B66] Xue G. Chen C. Lu Z. L. Dong Q. (2010). brain imaging techniques and their applications in decision-making research. Xin Li Xue Bao 42, 120–137. 10.3724/SP.J.1041.2010.00120 20376329 PMC2849100

[B67] Ye J. C. Tak S. Jang K. E. Jung J. Jang J. (2009). NIRS-SPM: statistical parametric mapping for near-infrared spectroscopy. Neuroimage 44, 428–447. 10.1016/j.neuroimage.2008.08.036 18848897

